# Prevalence of Alloimmunization Among Patients With Transfusion-Dependent Thalassemia and Sickle Cell Disease in Salmaniya Medical Complex, Bahrain

**DOI:** 10.7759/cureus.113206

**Published:** 2026-07-23

**Authors:** Jaffer Altooq, Zainab Sultan, Zainab Harb, Fatema Abdulla, Maryam AlOmran, Sharaf Almeshal

**Affiliations:** 1 Hematology, Salmaniya Medical Complex, Manama, BHR; 2 Hematology, Royal College of Surgeons in Ireland, Busaiteen, BHR; 3 Histopathology, Salmaniya Medical Complex, Manama, BHR

**Keywords:** alloimmunization, bahrain, blood transfusion, sickle cell disease, thalassemia

## Abstract

Background

Red blood cell (RBC) alloimmunization is a significant complication among patients with transfusion-dependent thalassemia and sickle cell disease (SCD), potentially leading to difficulties in crossmatching, delayed hemolytic transfusion reactions, and hyperhemolysis. In Bahrain, the prevalence of RBC alloimmunization among patients with hemoglobinopathies had not been previously reported.

Objective

To determine the prevalence of RBC alloimmunization among patients with transfusion-dependent thalassemia and SCD in Bahrain and to compare findings with published regional and international data.

Methods

A retrospective, cross-sectional study was conducted at Salmaniya Medical Complex (SMC), Bahrain. A total of 311 transfusion-dependent patients, 203 with SCD and 108 with thalassemia, registered between 2016 and 2025 were included. Clinical and laboratory data were extracted from electronic medical records. Patients with a positive indirect antiglobulin test (IAT) showing a consistent reaction strength of 2+ or greater were considered alloimmunized. Descriptive statistics were used to analyze demographic variables and determine the prevalence of alloimmunization. A chi-square test was used to compare alloimmunization rates between the SCD and thalassemia groups. The rates observed in this study were also compared with those reported in other countries using chi-square analysis. A p-value < 0.05 was considered statistically significant.

Results

The overall prevalence of RBC alloimmunization was 14.8%. Alloimmunization was significantly more prevalent among patients with SCD, with 41 out of 203 (20.2%) testing positive, compared to only 5 out of 108 (4.6%) patients with thalassemia (p < 0.001). Among the 46 alloimmunized patients, 58.7% were male, and 41.3% were female, reflecting the sex distribution of the study population. Blood group O was the most common (54.3%), and 95.5% of patients were Rh-positive.

Conclusion

The alloimmunization rates observed in this study are consistent with the rates reported in other Middle Eastern countries. Despite extended phenotypic crossmatching, patients with SCD had a significantly higher alloimmunization rate than patients with thalassemia, likely due to disease-related immune activation and differences in transfusion protocols. Adoption of molecular RBC genotyping and comprehensive antibody identification for all alloimmunized patients is recommended to improve transfusion safety in this population.

## Introduction

Sickle cell disease (SCD) and thalassemia are inherited hemoglobin disorders that predominantly affect individuals of African, Middle Eastern, Mediterranean, and Indian descent and represent a major global health burden [1]. For many patients, particularly those with severe symptoms, regular red blood cell (RBC) transfusions remain a cornerstone of management by correcting chronic anemia and helping to prevent serious complications such as stroke, acute chest syndrome, and progressive organ damage [2]. However, repeated transfusions expose patients to numerous foreign RBC antigens that can stimulate the immune system to produce alloantibodies directed against them. This process, known as RBC alloimmunization, is a well‑recognized complication in patients with transfusion-dependent SCD and thalassemia and can significantly complicate transfusion therapy. RBC alloimmunization can lead to difficulties in finding compatible blood units, delayed hemolytic transfusion reactions, and, in severe cases, life-threatening hyperhemolysis [3].

Current guidelines, including those from the American Society of Hematology (ASH) and the International Collaboration for Transfusion Medicine Guidelines, recommend prophylactic matching for ABO, Rh (C, c, D, E, e), and Kell (K) antigens for all patients with SCD and thalassemia, even in the absence of preexisting alloantibodies [4]. Despite these measures, alloimmunization still occurs. Several factors explain this persistent risk. First, conventional serologic phenotyping may fail to detect variant *Rh *alleles, particularly partial *RHD* and *RHCE* alleles, which are common in individuals of African descent, leading to apparent Rh-matched transfusions that are in fact immunologically mismatched [5]. A recent study found clinically significant genotype-phenotype discrepancies in over 20% of patients with SCD, and partial *Rh* alleles in 17% of cases [5]. Second, standard matching protocols do not extend to other clinically important blood group systems such as Duffy (Fy), Kidd (Jk), and MNS (S/s), leaving patients vulnerable to sensitization against these antigens [6]. Third, in Europe and the USA, recipients of African or Middle Eastern descent are at an increased risk of antigen mismatch because donor pools are predominantly of European origin and red cell antigen profiles differ significantly between these populations [7]. Beyond transfusion-related variables, several patient-related factors have been associated with increased alloimmunization susceptibility. These include older age, female sex, older age at first transfusion, higher cumulative transfusion exposure, history of splenectomy, presence of RBC autoantibodies, and an underlying pro-inflammatory or immunogenetic predisposition [8].

The reported prevalence of alloimmunization varies considerably across populations. A systematic review of 41 cohort studies involving over 9,000 patients with thalassemia reported a pooled prevalence of 11.4%, with antibodies against Rh and Kell antigens accounting for approximately 78% of all alloantibodies identified [9]. In SCD, alloimmunization rates have been reported to range from 8% to 36%, depending on the study population and transfusion practices employed. A recent systematic review focusing specifically on Middle Eastern countries found mean alloimmunization prevalence rates of 13.0% in thalassemia and 14.0% in SCD, with considerable heterogeneity among individual studies [8]. Individual cohort reports range from as low as 2-8% in some African and South Asian centers to over 30% in Omani patients with SCD [10-12]. The aim of this study was to determine the prevalence of RBC alloimmunization among patients with transfusion-dependent thalassemia and SCD in Bahrain and to compare the observed alloimmunization rate with published data from other regional and international studies.

## Materials and methods

Study design and setting 

This retrospective, cross-sectional study was conducted at Salmaniya Medical Complex (SMC), the largest tertiary care hospital in Bahrain, after obtaining approval from the Research Committee for Government Hospitals (no. 76-310725).

Inclusion and exclusion criteria 

This study included patients of all ages with transfusion-dependent thalassemia or SCD who were registered in the hospital blood bank between 2016 and 2025. Patients with transfusion-dependent thalassemia received simple transfusions every 2-4 weeks, whereas patients with transfusion-dependent SCD received exchange transfusions every 3-8 weeks. Patients were eligible for inclusion if they had undergone at least one indirect antiglobulin test (IAT) during the study period. Patients were excluded if their records were incomplete, specifically for cases in which no IAT was performed between 2016 and 2025. A total of 311 patients, 203 with SCD and 108 with thalassemia, met the eligibility criteria and were included in this study. 

Data collection 

Clinical and laboratory data were extracted from I-SEHA, the hospital’s electronic medical records system. The collected data included demographic characteristics (age, gender, ABO blood group, and Rh factor) and alloimmunization status. Patients who had a positive IAT with a consistent reaction strength of 2+ or greater were considered alloimmunized. Patients with consistently negative or weakly positive IAT reactions were not classified as alloimmunized.

Laboratory methods

Whole blood samples were collected in ethylenediaminetetraacetic acid (EDTA) tubes, transported to the blood bank under standard conditions, and processed within 24 hours of collection.

Pre-transfusion testing throughout the study period followed SMC blood bank policy and included ABO and RhD typing, antibody screening, and compatibility testing. Antibody screening was performed for all patients using an IAT-based gel column agglutination method with commercially prepared screening cells (0.8% Selectogen; Ortho-Clinical Diagnostics, Inc., NJ, USA). A direct antiglobulin test (DAT) was also performed as part of the routine pre-transfusion workup.

Antibody identification to determine specific alloantibody types and frequencies was performed only on a limited subset of patients with reactive screens using standard reagent red cell panels (0.8% Resolve Panel A; Ortho-Clinical Diagnostics, Inc., NJ, USA). The decision to perform antibody identification was either based on the clinician’s request or at the discretion of the transfusion medicine and blood banking physician.

Statistical analysis

Descriptive statistical analysis was performed using IBM SPSS version 29 (released 2022; IBM Corp., Armonk, NY). The software was used to determine the frequencies and percentages of demographic variables and the prevalence of alloimmunization within the studied population. Chi-square tests were then used to compare alloimmunization rates between the SCD and thalassemia groups, and to compare the rates observed in this study with those reported in other countries. A p-value of less than 0.05 was considered statistically significant for all comparisons.

## Results

A total of 311 transfusion-dependent patients were included, comprising 203 (65.3%) with SCD and 108 (34.7%) with thalassemia. The overall age range was 15-84 years, with a mean age of 38.33 years. Of the total cohort, 185 (59.5%) were male, and 126 (40.5%) were female (Table 1).

**Table 1:** Gender distribution among transfusion-dependent patients with sickle cell disease and thalassemia

Diagnosis		Gender		Total
		Female	Male	
Sickle cell disease		73 (57.9%)	130 (70.3%)	203 (65.3%)
Thalassemia		53 (42.1%)	55 (29.7%)	108 (34.7%)
Total		126 (100.0%)	185 (100.0%)	311 (100.0%)

ABO and Rh typing revealed that blood group O was the most prevalent at 169 (54.3%), followed by B at 67 (21.5%), A at 65 (20.9%), and AB at 10 (3.2%). The vast majority of patients were Rh-positive, 297 (95.5%), while 14 (4.5%) were Rh-negative. Among the 14 Rh-negative individuals, 7 (50.0%) belonged to blood group O, 6 (42.9%) to group B, and 1 (7.1%) to group A, with none in group AB (Table 2).

**Table 2:** Distribution of ABO blood groups and Rh factor among the study participants

Blood group (ABO)		Rh factor		Total
		Negative	Positive	
A		1 (7.1%)	64 (21.5%)	65 (20.9%)
AB		0 (0.0%)	10 (3.4%)	10 (3.2%)
B		6 (42.9%)	61 (20.5%)	67 (21.5%)
O		7 (50.0%)	162 (54.5%)	169 (54.3%)
Total		14 (100.0%)	297 (100.0%)	311 (100.0%)

The overall prevalence of red cell alloimmunization, as determined by the IAT, was 46 (14.8%). When stratified by disease type, alloimmunization was significantly more frequent among patients with SCD, with 41 out of 203 (20.2%) testing positive for alloantibodies, compared with only 5 out of 108 (4.6%) among patients with thalassemia. A statistically significant difference between the two disease groups was confirmed using chi-square analysis (p < 0.001). Among the 46 alloimmunized patients, 27 (58.7%) were male, and 19 (41.3%) were female. SCD accounted for the majority of alloimmunized cases at 41 (89.1%), of whom 24 were male and 17 were female. The remaining five (10.9%) alloimmunized patients had thalassemia, comprising three males and two females. These findings suggest a slightly higher proportion of alloimmunization among males, although both sexes were clearly affected.

## Discussion

Compared to other countries, Bahrain’s SCD alloimmunization rate was intermediate, lower than Oman but higher than Saudi Arabia, Nigeria, Tanzania, and India (Table 3).

**Table 3:** Comparison between the SCD alloimmunization rate in Bahrain and rates reported in other countries p-values were calculated using the chi-square test. SCD: sickle cell disease

Study location	Alloimmunized patients/total	Alloimmunization rate (%)	P-value
Current study	41/203	20.2%	
Oman [12]	42/133	31.6%	0.018
Al-Ahsa, Saudi Arabia [13]	13/78	16.7%	0.50
Central India [10]	3/140	2.15%	<0.001
Tanzania [11]	17/200	8.5%	<0.001
Abuja, Nigeria [14]	26/205	17.6%	0.50

However, the alloimmunization rate among patients with thalassemia in Bahrain was lower than the rates reported in Oman, Saudi Arabia, Egypt, and India (Table 4).

**Table 4:** Comparison between the thalassemia alloimmunization rate in Bahrain and rates reported in other countries p-values were calculated using the chi-square test.

Study location	Alloimmunized patients/total	Alloimmunization rate (%)	P-value
Current study	5/108	4.6%	
Oman [12]	26/129	20%	<0.001
Al-Ahsa, Saudi Arabia [13]	34/284	11.8%	0.03
North India [15]	17/255	6.6%	0.46
Cairo, Egypt [16]	36/200	18%	0.001

A notable finding of this study was the significant difference in alloimmunization rates between the two disease groups. Patients with SCD had a significantly higher prevalence of 20.2% compared to only 4.6% in patients with thalassemia. This disparity is consistent with the findings from several other centers. Kuriri et al. (2023) reported alloimmunization rates of 16.7% in SCD versus 11.97% in thalassemia in the Al-Ahsa region of Saudi Arabia [13]. A study from Oman documented even more pronounced differences, with rates of 31.6% in SCD and 20% in thalassemia [12]. The higher alloimmunization rate in patients with SCD has been attributed to the pro-inflammatory state inherent to SCD, which can make the immune system more likely to produce alloantibodies. Additionally, the intermittent and episodic nature of transfusions in SCD compared to the regular, scheduled transfusions in thalassemia may further increase the risk of alloimmunization [17].

The slightly higher prevalence of alloimmunization among males (58.7%) compared to females (41.3%) in our alloimmunized group is an interesting observation, though the difference should be interpreted cautiously, given the overall male predominance in the study population (59.5%). The published literature is inconsistent on the role of sex as a risk factor. Some studies have reported higher rates in females, potentially due to previous pregnancy-related sensitization, while others, including Al-Allawi et al. (2025), identified female sex as a significant risk factor in several Middle Eastern studies [8,18]. The male predominance observed in our cohort may simply reflect the sex distribution of the underlying study population rather than a true biological predisposition. Moreover, this study did not account for other confounding risk factors such as cumulative transfusion burden, age at first transfusion, splenectomy status, or pregnancy history. Therefore, no definitive conclusions can be drawn regarding sex as an independent risk factor for alloimmunization.

Regarding blood group distribution, blood group O was the most prevalent in our cohort (54.3%), and the overwhelming majority (95.5%) were Rh-positive. This distribution is reflective of the blood group frequencies reported in Arab populations. In settings where the donor pool is ethnically similar to the recipient population, the antigenic mismatch that drives alloimmunization may be reduced. Gader et al. (2008) demonstrated this in a Saudi Arabian study, where none of the 13 patients who received blood exclusively from Arab donors developed alloantibodies, compared to a 22% alloimmunization rate when multi-ethnic donor blood was used [19].

The overall alloimmunization rate of 14.8%, and particularly the rate of 20.2% in patients with SCD, highlights the ongoing challenge of alloimmunization at SMC despite the existing matching protocols. Currently, SMC employs extended phenotypic crossmatching for patients with SCD and provides Rh and Kell antigen-matched transfusions for patients with thalassemia. The lower alloimmunization rate observed in patients with thalassemia (4.6%) compared to patients with SCD (20.2%) may, in part, reflect the protective effect of Rh and Kell matching in this group. Franchini et al. (2019) estimated that matching for Rh and Kell antigens alone could reduce alloimmunization by approximately 80% [9]. Similarly, a meta-analysis by Sugrue et al. (2024) found that adding c, E, and K matching to standard ABO/RhD matching reduced alloimmunization rates from 6.2% to 1.9% [20].

The persistently high alloimmunization rate among patients with SCD despite extended phenotypic matching is a concern that warrants further investigation. The chronic inflammatory state inherent to SCD and Rh antigen variant alleles, which are common in populations of African and Middle Eastern descent, can lead to alloimmunization even when conventional serologic matching appears compatible [17,21]. Al-Allawi et al. (2025) specifically highlighted the need for more research on Rh variants in the Middle East, as these may contribute to residual alloimmunization despite phenotypic matching [8]. The adoption of molecular genotyping for Rh variants, in addition to the current serologic phenotyping, could help identify patients at risk for alloimmunization due to variant antigen expression and further improve donor-recipient matching [5].

Strengths

This study has several notable strengths. It is the first to report the prevalence of RBC alloimmunization among patients with transfusion-dependent thalassemia and SCD in Bahrain, addressing an important gap in the regional literature. The study also included a relatively large cohort of 311 patients drawn from the largest tertiary care hospital in Bahrain. Moreover, the study covered a broad nine-year period (2016-2025), allowing for a more robust assessment of alloimmunization prevalence. In addition, the use of a standardized IAT-based gel column agglutination method with a clearly defined alloimmunization threshold ensured methodological consistency across all patients throughout the study period. Finally, the inclusion of both patients with SCD and thalassemia enabled a direct statistical comparison of alloimmunization rates between the two disease groups.

Limitations

This study has several limitations. First, data on alloantibody types (e.g., anti-K, anti-E, anti-C) and their frequencies were not available for the majority of alloimmunized patients and were therefore excluded from this study. This is a significant gap, as knowledge of the predominant antibody types is essential for guiding transfusion matching strategies. Second, data on risk factors of alloimmunization, such as cumulative transfusion exposure, age at first transfusion, splenectomy, pregnancy history, and Rh variant status, were not collected or analyzed. As a result, we were unable to assess the contribution of these risk factors to alloimmunization. Third, the retrospective design of the study relied on electronic medical records, which may have contained missing or incomplete information. In addition, the frequency of IAT testing differed across patients, as some underwent testing only once, whereas others had repeated IAT tests. This may have introduced variability in the detection of alloimmunization over time. Finally, patients with weak (1+) IAT reactions were not classified as alloimmunized because most of these reactions were transient and became negative on repeat testing, suggesting false-positive results. Excluding weakly positive IAT reactions likely improved specificity but may have slightly reduced sensitivity, leading to an underestimation of the true prevalence of alloimmunization.

## Conclusions

This study shows that RBC alloimmunization remains an important transfusion-related complication among patients with thalassemia and SCD at SMC, Bahrain. The alloimmunization rates observed in this study are consistent with rates reported in other Middle Eastern countries. Alloimmunization was substantially more prevalent among patients with SCD than among patients with thalassemia, likely reflecting differences in disease-related immune activation and transfusion protocols. The low rate in patients with thalassemia suggests that current Rh and Kell matching offers adequate protection in this group. In contrast, the rate in patients with SCD remains elevated despite the use of extended phenotypic crossmatching. The absence of comprehensive alloantibody identification data is a notable limitation. Future studies should ensure complete antibody identification for all patients with positive screens. Investigation of Rh variant alleles and consideration of molecular red cell genotyping as a complement to serologic phenotyping, particularly for patients with SCD, are also warranted to improve transfusion safety in this population.
